# The prognostic potential of circulating BDNF levels and its polymorphisms in age-related cognitive impairment and neurodegeneration

**DOI:** 10.3389/fnagi.2026.1901527

**Published:** 2026-07-27

**Authors:** Tatiana V. Tulupova, Maria V. Vedunova, Elena V. Mitroshina

**Affiliations:** Institute of Biology and Biomedicine, National Research Lobachevsky State University of Nizhny Novgorod, Nizhny Novgorod, Russia

**Keywords:** biomarkers, brain-derived neurotrophic factor (BDNF), cognitive impairment, neurodegenerative diseases, Val66Met polymorphism

## Abstract

Brain-derived neurotrophic factor (BDNF) is essential for neuronal survival, synaptic plasticity, and cognitive function. Age-related decline in BDNF signaling has been implicated in the pathogenesis of Alzheimer’s disease, Parkinson’s disease, and mild cognitive impairment. However, the prognostic value of circulating BDNF and its genetic variants remains controversial due to inconsistent findings across studies. This review synthesizes current evidence on peripheral BDNF levels and the Val66Met polymorphism as potential biomarkers of age-related cognitive decline and neurodegeneration. We critically analyze biological mechanisms linking BDNF to neurodegeneration, including its interaction with amyloid-beta and tau pathology. We further examine factors underlying discrepant results: demographic characteristics, comorbidities, lifestyle factors, pharmacological interventions, and methodological variability. Despite these challenges, BDNF remains a promising diagnostic factor. Nevertheless, standardizing preanalytical protocols and accounting for patient heterogeneity are essential to unlock its diagnostic potential.

## 1 Introduction

Increasing life expectancy and the progressive growth of the elderly population have led to a rise in the prevalence of age-related cognitive impairment and neurodegenerative disorders, including Alzheimer’s disease and Parkinson’s disease. Early diagnosis and prediction of disease progression remain challenging, as neuronal dysfunction and the initial pathological processes often precede the onset of clinical manifestations, while early symptoms are frequently non-specific. Therefore, the identification and validation of accessible, minimally invasive biomarkers capable of detecting pathological processes at the preclinical stage, predicting the rate of cognitive decline, and assessing the efficacy of potential neuroprotective therapies are of considerable importance. Peripheral biomarkers measurable in serum or plasma represent the most practical option for routine clinical use; however, reliable peripheral biomarkers of neurodegeneration remain limited.

Over the past decade, accumulating evidence has linked alterations in the expression of neurotrophic factors, particularly brain-derived neurotrophic factor (BDNF), with the development of neurodegenerative processes (Ventriglia et al., 2013; Ng et al., 2019). BDNF plays a critical role in the development of both the central and peripheral nervous systems and is essential for neuronal maturation, survival, and functional maintenance. In addition, BDNF promotes synaptogenesis and regulates synaptic transmission and plasticity (Toader et al., 2025).

Alterations in BDNF signaling pathways and changes in its concentration affect memory and cognitive function in a range of age-related cognitive disorders, including Alzheimer’s disease and mild cognitive impairment (Miranda et al., 2019). However, evidence regarding the relationship between peripheral BDNF levels and neurodegenerative diseases remains inconsistent. Several studies have reported reduced peripheral BDNF concentrations in patients with Alzheimer’s disease and mild cognitive impairment compared with healthy controls (Ng et al., 2019; Yasutake et al., 2006), whereas others have demonstrated elevated BDNF levels in patients with Alzheimer’s disease (Qian et al., 2022).

These discrepancies may be attributable to multiple factors, including demographic characteristics (age and sex), comorbid conditions, medication use, lifestyle factors, and methodological differences among studies, such as BDNF detection methods, the type of biological material analyzed (serum or plasma), and other study-specific parameters. In addition, genetic polymorphisms of the *BDNF* gene, particularly the *Val66Met* variant, may influence intracellular transport and secretion of the protein, thereby contributing to individual susceptibility to accelerated cognitive aging.

In this review, we summarize current evidence regarding the prognostic significance of peripheral BDNF and evaluate its clinical utility as a biomarker of cognitive aging and neurodegenerative processes.

## 2 BDNF: functions and roles

Neurotrophic factors are signaling proteins involved in key cellular processes regulating neuronal maturation, differentiation, survival, and synaptic plasticity within the central nervous system (CNS). Among them, brain-derived neurotrophic factor (BDNF) is one of the most extensively studied. Within the brain, BDNF is expressed by glutamatergic neurons and glial cells of the hippocampus, cerebral cortex, substantia nigra, amygdala, cerebellum, and other regions. Outside the nervous system, BDNF is present in platelets, leukocytes, lymphocytes, endothelial cells, and vascular smooth muscle cells (Colucci-D’Amato et al., 2020).

Synaptic and neuronal plasticity are essential characteristics of a structurally and functionally integrated brain. Neuronal plasticity encompasses several processes, including neurogenesis – the generation of new neurons within proliferative zones – morphological remodeling of mature neurons, synaptogenesis, and the growth and branching of neuronal processes. BDNF has been shown to play a direct role in these processes and, at the physiological level, is critically involved in learning and memory (Cunha et al., 2010), as well as in neural tissue regeneration following injury and protection against neuronal damage (Liu et al., 2020; Guo et al., 2024).

The functions and biological role of BDNF in the regulation of physiological processes within the CNS should be considered in the context of interactions between its isoforms and their respective receptors. During maturation, the precursor protein preproBDNF undergoes cleavage of its signal peptide within the Golgi apparatus, resulting in the formation of proBDNF, a group of isoforms retaining the pro-domain. The mature BDNF isoform (mBDNF) is subsequently generated through proteolytic cleavage of the pro-domain by intracellular endopeptidases, intravesicular convertases, extracellular plasmin, and matrix metalloproteinases 2 and 9 (MMP2 and MMP9). Both proBDNF and mBDNF are expressed in the brain; however, their relative abundance varies across developmental stages and postnatal life, with mBDNF predominating in adulthood. These isoforms are believed to exert opposing biological effects.

The proBDNF/p75NTR/sortilin complex initiates signaling cascades leading to activation of c-Jun N-terminal kinase (JNK), a pathway associated with neuronal apoptosis. In contrast, the binding of mature BDNF to p75NTR activates signaling mediated by receptor-interacting serine/threonine-protein kinase 2 (RIP2) and TNF receptor-associated factor 6 (TRAF6), resulting in NF-κB activation. This pathway supports neuronal survival and maintenance (Kowiański et al., 2018).

The TrkB receptor, a member of the tyrosine kinase receptor family, exhibits the highest affinity for mBDNF. Its full-length isoform is expressed predominantly on neuronal membranes and is not detected outside the CNS. In contrast, truncated receptor isoforms, including TrkB.T1, are expressed by glial cells and are present on the surface of cells in the heart, kidneys, lungs, and pancreas of adult humans (Tessarollo and Yanpallewar, 2022). Binding of BDNF to TrkB activates the PI3K/Akt, MAPK/ERK, and PLC-γ signaling pathways, which regulate numerous cellular processes, including neuronal survival and apoptosis, neuronal differentiation, dendritic growth and branching, calcium mobilization, cytoskeletal remodeling, synapse formation, and synaptic plasticity (Soman et al., 2025; Harun et al., 2025).

BDNF plays a critical role in the development and maintenance of synaptic contacts and neural networks. Synaptic plasticity is the key cellular mechanism underlying the regulation of neural network activity and the implementation of cognitive brain functions. BDNF influences both functional and structural aspects of synaptic plasticity (Kowiański et al., 2018).

The most well-studied form of synaptic plasticity responsible for memory formation is long-term potentiation (LTP), a sustained increase in synaptic efficacy following high-frequency stimulation. Long-term potentiation can be divided into two phases: early long-term potentiation (E-LTP) and late long-term potentiation (L-LTP). The early phase is characterized by modulation of synapse structure, changes in the number and subunit composition of receptors at the synaptic membrane, and alterations in the quantity of neurotransmitters released. The late phase, in turn, is associated with changes in gene expression and protein synthesis, as well as microRNAs (Kowiański et al., 2018; Minichiello, 2009).

These processes involve signaling cascades triggered by the binding of mBDNF to TrkB. For example, at the presynaptic membrane, the MAPK/ERK cascade leads to phosphorylation of synapsin I, resulting in the release of glutamate-containing synaptic vesicles from the reserve pool and subsequent neurotransmitter exocytosis (Tyler and Pozzo-Miller, 2001; Jovanovic et al., 2000). TrkB signals also activate Ca^2+^/calmodulin-dependent kinase II (CaMKII) and protein kinase C (PKC), which phosphorylate subunits of AMPA-type glutamate receptors (AMPARs), increasing their synaptic delivery, thereby organizing active signaling zones at the postsynaptic membrane (Caldeira et al., 2007).

At the structural level, the involvement of mBDNF in synaptic plasticity is mediated by changes in dendritic spine morphology, cytoskeletal reorganization, and the formation of new synapses. The molecular cascade triggered by BDNF upon binding to TrkB leads to activation of LIM kinase, which phosphorylates and inhibits cofilin, a protein that depolymerizes F-actin (Dong et al., 2012). BDNF, via the PI3K/Akt cascade, stimulates the transport and clustering of the major scaffolding protein PSD-95 (Yoshii and Constantine-Paton, 2007). The BDNF–TrkB signaling pathway activates several Rho-GTPase proteins that regulate actin filament dynamics and are responsible for dendritic branching (Cheung et al., 2007).

The BDNF–TrkB signaling pathway is involved in the regulation of protein synthesis at postsynaptic terminals, stabilizing synaptic changes and underpinning late long-term potentiation, which is necessary for memory formation (Gonzalez et al., 2016). BDNF locally triggers the translational machinery through activation of translation initiation factors, for example, 4EBP1 (Takei et al., 2004). BDNF triggers CREB-induced expression of genes encoding regulators of synaptic activity, such as Arc (Ying et al., 2002; Zheng et al., 2009), several synaptic vesicle proteins, including vesicular glutamate transporters (Melo et al., 2013) and proteins of the translational machinery, including eIF4E (Kanhema et al., 2006) and eEF2 (Manadas et al., 2009).

The crucial role of proBDNF in the regulation of another process involved in synaptic plasticity – long-term depression (LTD) – should also be noted (Yang et al., 2014; Woo et al., 2005). proBDNF–p75NTR-mediated signaling reduces the number of dendritic branches and spine formation in granule neurons of the dentate gyrus, and a similar effect on spine density was observed in CA1 pyramidal neurons (Yang et al., 2014).

Thus, long-term potentiation ensures the reorganization and stabilization of synapses, underpinning the strengthening of neural networks required for learning and memory processes. Together with long-term depression, these processes establish the balance necessary for brain plasticity and the efficiency of learning and memory. Neurodegenerative processes in the brain are accompanied by neuronal loss and the decline of synaptic and neuronal plasticity, partly due to reduced BDNF levels. BDNF deficiency in aging and neurodegeneration is associated with impaired synaptic plasticity, which manifests as progressive deterioration of memory and learning ability. The relationship between cognitive decline and decreased BDNF concentrations supports the consideration of BDNF as a promising diagnostic marker.

Peripheral functions of BDNF include the regulation of cardiovascular homeostasis through its effects on platelet activation and aggregation, as well as the secretion of inflammatory and angiogenic cytokines (Boukhatem et al., 2021). BDNF also plays an important role in the regulation of cardiac contractility, promotes cellular survival and tissue repair following injury, including myocardial infarction, and contributes to the regulation of angiogenesis (Fulgenzi et al., 2015; Kermani and Hempstead, 2019). Muscle-derived BDNF supports motor neuron survival and participates in skeletal muscle regeneration and metabolic regulation (Yang et al., 2019; Rentería et al., 2022).

BDNF additionally plays a significant role in the regulation of inflammatory responses within the CNS. It exerts anti-inflammatory effects on microglial cells by reducing their activation (Wu et al., 2020; Charlton et al., 2023) and has been shown to slow the progression of neuronal damage in multiple sclerosis. Furthermore, BDNF promotes the maturation, proliferation, and differentiation of T and B lymphocytes, acting as a protective and supportive factor in certain autoimmune diseases (Wang and Tian, 2021).

Given the critical role of BDNF in CNS function, it has attracted considerable attention as a potential neuroprotective factor in neurodegenerative disorders. Age is one of the principal contributors to cognitive decline, as advancing age is associated with a substantial reduction in gray matter volume, particularly due to accelerated hippocampal atrophy. Neurodegenerative disorders, including Alzheimer’s disease, Parkinson’s disease, Huntington’s disease, and amyotrophic lateral sclerosis (ALS), are characterized by neuronal loss and impaired neuroplasticity, resulting in cognitive dysfunction ranging from deficits in memory, attention, and thinking to the development of dementia. Alterations in circulating BDNF levels may reflect early age-related changes in its expression within the brain. Therefore, the assessment of peripheral BDNF concentrations may have potential value as a prognostic marker of neurodegenerative processes and as an indicator of disease progression (Pisani et al., 2023).

## 3 Peripheral BDNF levels as a biomarker of cognitive impairment in aging and neurodegeneration

In recent years, numerous studies have investigated the relationship between circulating BDNF levels and cognitive dysfunction associated with aging and neurodegenerative diseases. Reduced BDNF concentrations have been reported in patients with Parkinson’s disease (PD), Alzheimer’s disease (AD), and mild cognitive impairment (Colucci-D’Amato et al., 2020; Alqahtani et al., 2025).

In animal models BDNF has been shown to cross the blood–brain barrier (Pan et al., 1998; Poduslo and Curran, 1996), and blood BDNF levels correlate with those in brain tissue and cerebrospinal fluid (Klein et al., 2011; Karege et al., 2002; Pillai et al., 2010). In patients with Alzheimer’s disease, postmortem cortical BDNF levels correlated with those in serum, and BDNF levels were associated with cognitive decline (Laske et al., 2006b).

Age-related increases in blood–brain barrier (BBB) permeability are well documented in numerous studies (Konig et al., 2025; Marques et al., 2013). Cerebral vascular network density decreases with age, and age-related BBB changes contribute to disrupted brain homeostasis and the development of various neurodegenerative diseases. Research indicates that anatomical alterations, such as reduced tight junction protein levels and pericyte degeneration or loss, may compromise BBB function (Costea et al., 2019). The progressive inflammatory state characteristic of the aging brain also significantly contributes to cerebrovascular dysfunction (Konig et al., 2025). As senescent endothelial cells accumulate in the BBB, their altered function compromises barrier integrity. These cells exhibit impaired barrier properties, degrade the extracellular matrix, and enhance transcytosis, resulting in increased barrier permeability (Real et al., 2024; Ting et al., 2023).

Cerebrovascular dysfunction and vascular pathology contribute to cognitive decline and neuronal death in Alzheimer’s disease, in addition to pathologies associated with amyloid-beta and tau proteins (Zlokovic, 2011; Wu et al., 2024). Beyond the aforementioned factors, BBB permeability is also influenced by sex, temperature, mental state, medication use, and physical activity (Zhao et al., 2022). Age-related BBB disruption may be associated with an exacerbated inflammatory milieu in the brain, impaired clearance of toxic proteins and metabolites, and consequently affecting cognitive function. Age-related cognitive decline may be related to both alterations in BDNF levels and compromised BBB integrity. However, the influence of numerous additional factors on both processes makes their analysis particularly challenging and requires in-depth studies to accurately assess their respective contributions to cognitive impairment.

Laske et al. (2006a) were among the first to suggest that peripheral BDNF levels may reflect the dynamic progression of Alzheimer’s disease. According to this hypothesis, elevated BDNF levels during the early stages of AD represent a compensatory neuroprotective response to neurotoxicity induced by the accumulation of β-amyloid and tau protein, whereas the subsequent decline in BDNF levels reflects the progression and extent of neurodegeneration. Indeed, β-amyloid accumulation has been shown to reduce BDNF synthesis, including through disruption of CREB phosphorylation, one of the key transcriptional regulators of *BDNF* expression (Rosa and Fahnestock, 2015). Impairment of BDNF/TrkB signaling promotes the accumulation of delta-secretase, an enzyme involved in amyloidogenic processing of amyloid precursor protein and in the abnormal aggregation of toxic Aβ_1–40_ and Aβ_1–42_ peptides. In addition, oligomeric Aβ_1–42_ markedly suppresses overall *BDNF* expression through selective inhibition of *BDNF* transcripts IV and V (Garzon and Fahnestock, 2007).

BDNF also counteracts the development of tau pathology by reducing the activity of glycogen synthase kinase 3β (GSK3β), the principal enzyme responsible for tau hyperphosphorylation and neurofibrillary tangle formation (Lei et al., 2023). Furthermore, BDNF activates autophagic pathways that may facilitate the clearance of hyperphosphorylated tau species. Conversely, tau overexpression leads to dysregulation of both *BDNF* expression and BDNF/TrkB signaling. Several studies have demonstrated an association between reduced BDNF levels and increased tau concentrations (Ginsberg et al., 2019). Thus, BDNF deficiency in Alzheimer’s disease is closely associated with the development of tau pathology.

Despite the mechanisms described above that contribute to reduced BDNF levels in Alzheimer’s disease, measurement of circulating BDNF has not yet demonstrated definitive diagnostic utility in individuals with cognitive impairment. As noted previously, the non-linear dynamics of *BDNF* expression may reflect the activation of compensatory protective mechanisms during the progression of neurodegeneration. Although most studies have reported an overall reduction in peripheral BDNF levels in patients with AD (Passaro et al., 2015; Janel et al., 2017), together with a positive correlation with worsening cognitive performance as the disease progresses, other investigations have found no significant differences in BDNF levels between patients with Alzheimer’s disease and cognitively healthy controls (Woolley et al., 2012; van den Bosch et al., 2021).

Furthermore, BDNF plays a critical role in the survival and function of dopaminergic neurons, protecting them from the detrimental effects of an acidic environment. At the cellular level, BDNF promotes dopamine synthesis and release, protects against oxidative stress, and regulates axonal transport (Toader et al., 2025). Several studies have reported that BDNF levels decline in Parkinson’s disease as the disease progresses (Huang et al., 2018; Khalil et al., 2016; Huang et al., 2021), and that this decline correlates with the development of cognitive impairment (Costa et al., 2015; Wang et al., 2024). At the same time, Huang et al. (2018) demonstrated that elevated serum BDNF concentrations were associated with earlier disease onset and greater symptom severity. Similarly, Ventriglia et al. (2013) reported increased BDNF levels in the advanced stages of Parkinson’s disease.

Thus, the available evidence regarding the utility of peripheral BDNF as a diagnostic biomarker remains highly inconsistent. Several factors may contribute to these discrepancies.

First, demographic characteristics, including age and sex, may influence BDNF levels. Numerous studies have demonstrated an age-related decline in circulating BDNF concentrations, regardless of the presence or severity of cognitive impairment (Ziegenhorn et al., 2007; Erickson et al., 2010; Driscoll et al., 2012). Some investigations have reported higher BDNF levels in women and have identified an association between declining BDNF concentrations and an increased risk of cognitive deterioration exclusively in female populations (Kim et al., 2025). For example, Shimada et al. (2014) demonstrated a reduction in BDNF levels only among women with Alzheimer’s disease who were carriers of the *APOE*ε*4* allele.

Second, lifestyle factors, including smoking, alcohol consumption, and physical activity, may affect BDNF concentrations. Physical exercise is considered one of the most effective approaches for maintaining cognitive function, as it supports neuroplasticity and contributes to memory formation and learning processes (Hwang et al., 2016; Walsh et al., 2020). Exercise stimulates neurogenesis through increased *BDNF* expression (Birinci et al., 2026). This effect is mediated by FNDC5, a protein produced by active skeletal muscle following activation of PGC-1α. FNDC5 is capable of crossing the blood–brain barrier and inducing *BDNF* expression. Increased BDNF levels have been observed both after a single bout of exercise and following regular physical training (Szuhany et al., 2015).

Chronic nicotine exposure has been shown to increase BDNF levels in the cerebral cortex and hippocampus. Experimental studies in animal models have provided insight into nicotine-induced alterations in *BDNF* expression. BDNF appears to play an important role in synaptic plasticity associated with the development of nicotine and drug dependence (Russo et al., 2009; Kim et al., 2019). Increased *BDNF* expression has been observed in brain regions involved in rewards processing, contributing to adaptive changes in neuronal structure and function and promoting oligodendrogenesis (Rullo et al., 2025).

Shimada et al. (2014) reported an association between smoking and elevated peripheral BDNF levels. Similarly, Weinstein et al. (2017) demonstrated that current smoking status, smoking duration, and the number of cigarettes smoked per day were associated with increased BDNF concentrations. Indeed, Abdelkhalek et al. (2022) reported elevated plasma BDNF levels in smokers, followed by a decline during smoking cessation. In contrast, other studies have found higher BDNF levels in individuals who had successfully quit smoking than in non-smokers (Bhang et al., 2010).

Furthermore, age-related physiological changes, psychiatric disorders, and the use of psychoactive medications may influence BDNF levels. For example, approximately 50% of patients with AD experience depression, a condition that, according to several studies, significantly affects BDNF concentrations in serum and plasma (Modrego, 2010; Botto et al., 2022). Numerous studies have reported reduced circulating BDNF levels in patients with depression (Molendijk et al., 2014; Kishi et al., 2017; Navarro et al., 2025). Moreover, Alvarez et al. (2014) demonstrated that alterations in BDNF levels may occur even in the presence of mild dysphoric symptoms indicative of subclinical depression.

Conversely, pharmacological treatment of depression is associated with a significant increase in circulating BDNF levels. Antidepressants enhance BDNF/TrkB signaling through mechanisms including TrkB autophosphorylation, increased CREB phosphorylation, and elevated *BDNF* mRNA expression (Fan et al., 2025). The increase in circulating BDNF observed during treatment with antidepressants and acetylcholinesterase inhibitors has led to the suggestion that BDNF may serve as a potential biomarker of therapeutic response to antidepressant treatment (Dreimüller et al., 2012). In Parkinson’s disease, particularly during the later stages, elevated serum BDNF levels have been associated with L-DOPA therapy, which is known to stimulate BDNF release, despite the fact that these patients initially exhibited lower BDNF levels than control subjects (Huang et al., 2018).

Altered BDNF levels have also been reported in patients receiving medications for age-related metabolic disorders, including lipid-lowering agents (Golden et al., 2010) and antidiabetic drugs (Eyileten et al., 2019; Sumbul-Sekerci et al., 2023). Shimada et al. (2014) found that reduced BDNF levels were associated with impaired glucose metabolism, whereas Weinstein et al. (2017) demonstrated that individuals with hypertension exhibited higher BDNF levels than those without hypertension. Furthermore, because circulating BDNF concentrations are closely related to the release of BDNF from platelets, failure to account for medications that affect platelet function, including anticoagulants and antihypertensive agents, may substantially influence study results.

Taken together, these findings underscore the importance of distinguishing alterations in circulating BDNF levels that are predictive of cognitive dysfunction from changes attributable to demographic characteristics, comorbid conditions, and therapeutic interventions.

A third factor contributing to the inconsistent findings regarding the diagnostic value of peripheral BDNF measurement is methodological variability among studies. As discussed above, BDNF is capable of crossing the blood–brain barrier from the brain; however, a substantial proportion of circulating BDNF is derived from platelets. Both serum and plasma are used for the assessment of peripheral BDNF concentrations. Notably, BDNF levels in serum are 100–200 times higher than those in plasma, a difference attributed to the release of BDNF from activated platelets during blood coagulation (Balietti et al., 2018; Thakkar and Acevedo, 2023). It has been demonstrated that serum BDNF concentrations, but not plasma concentrations, correlate with serotonin and β-thromboglobulin levels, both of which are markers of platelet activation (Laske et al., 2006a).

Furthermore, fluctuations in BDNF levels may reflect physiological or pharmacologically induced changes in platelet counts rather than age-related cognitive impairment. To reduce the contribution of platelet-derived BDNF, Ng (2025) proposed a modified protocol for sample preparation. Consequently, the simultaneous assessment of BDNF in both plasma and serum may represent the optimal approach. This strategy minimizes the confounding effects of platelet activation while enabling the evaluation of two distinct pools of BDNF: the “active” fraction (plasma BDNF), which primarily reflects freely circulating BDNF of cerebral origin, and the “reserve” fraction (serum BDNF), which is released upon platelet activation and may serve as a long-term biomarker.

It should also be noted that variability in the analytical performance of commercially available enzyme-linked immunosorbent assay (ELISA) kits may contribute substantially to the heterogeneity of published findings. Some assay systems do not exhibit absolute specificity for mature BDNF and may cross-react with its precursor and functional antagonist, proBDNF, thereby complicating the interpretation of results (Gao et al., 2022; Ng, 2025). Previous studies have shown that assay kits produced by R&D Systems and Aviscera Bioscience demonstrate greater selectivity for both BDNF isoforms than other commercially available kits and do not exhibit cross-reactivity (Polacchini et al., 2015). Among the kits evaluated in a recent comparative study, the R&D Systems kit (#DBD00) demonstrated the highest specificity for mature BDNF, although a cross-reactivity rate of 9.4% was still observed. At the same time, this kit showed high sensitivity and specificity for the quantification of proBDNF, making it possible to apply an alternative analytical approach based on calculating the difference between total BDNF and proBDNF concentrations (Olivas-Martínez et al., 2025).

The interaction of BDNF isoforms with specific receptors regulates various molecular processes that maintain a dynamic balance between excitatory and inhibitory effects on neuronal function. Signal cascades triggered by pro-BDNF and m-BDNF determine neuronal fate and the physiological functions of the brain, its development, and recovery after injury (Miranda et al., 2019). It is known that the pro-BDNF/m-BDNF ratio varies depending on the stage of brain development. In the early postnatal period, higher concentrations of pro-BDNF are observed, whereas m-BDNF predominates in adulthood. Pro-BDNF is considered an important factor in the elimination of damaged or malfunctioning neurons, retraction of growth cones and dendritic spines – processes essential for remodeling neural connections and promoting long-term depression (Kowiański et al., 2018; Sun et al., 2012). The mature form, in contrast, exerts neuroprotective functions, enhances neuronal development, and supports the formation of efficient synaptic connections and networks.

Some researchers have noted age-associated and pathology-associated changes in the pro-BDNF/m-BDNF ratio. Rodent models have shown that pro-BDNF accumulates in the hippocampus of aged animals, while m-BDNF levels remain stable (Buhusi et al., 2017; Perovic et al., 2013). Other studies have reported decreased m-BDNF and increased pro-BDNF levels in the dorsal and ventral hippocampus and prefrontal cortex of aged rats (Calabrese et al., 2013). Cade et al. (2023) demonstrated age-related decreases in m-BDNF in the hippocampus and increases in pro-BDNF in the prefrontal cortex and brainstem, with these trends observed before mice reached old age (9–12 months). In the study by Buhusi et al. (2017), elevated hippocampal pro-BDNF levels in aged rodents led to spatial memory impairments, and pro-BDNF infusion into the hippocampus negatively affected performance on memory and recognition tasks that animals had previously performed well on. Elevated pro-BDNF levels have been observed in the cerebrospinal fluid of Alzheimer’s disease patients (Fleitas et al., 2018), as well as in serum (Li et al., 2023), and correlated with cognitive impairment scores (Mini-Mental State Examination Score, MMSE).

Alterations in the processing of pro-BDNF into its mature form may be a contributing factor of the pro-BDNF/m-BDNF imbalance in the development of age-related cognitive impairment and neurodegeneration (Pisani et al., 2023; Alqahtani et al., 2025). It is known that the expression and levels of tissue plasminogen activator (tPA) decrease, while plasminogen activator inhibitor-1 (PAI-1) increases with age in the brains of wild-type mice, transgenic mice carrying the amyloid precursor protein and presenilin-1 (APP/PS1) genes, and in Alzheimer’s disease patients (Bi Oh et al., 2015; Liu et al., 2011). Using mouse models of Alzheimer’s disease as well as models of accelerated aging, it has been shown that plasmin and tPA levels in the hippocampus are reduced (Cai et al., 2020), while increased soluble Aβ led to elevated PAI-1 levels, which regulates plasmin-dependent pro-BDNF processing (Rodriguez et al., 2023). Comparable observations of elevated PAI-1 along with reduced tPA activity have been found in brain samples from Alzheimer’s disease patients (Fabbro and Seeds, 2009; Jiang et al., 2023), which may be associated with cognitive decline (Angelucci et al., 2019). ApoE is an important regulator of BDNF, affecting its gene transcription, maturation, and protein secretion. The ApoE ε4 isoform, one of the most well-studied risk factors for Alzheimer’s disease, suppresses BDNF transcription and also interferes with the cleavage and secretion of pro-BDNF and its conversion to the mature form (Sen et al., 2017). In addition, increased pro-BDNF levels may be related to its enhanced resistance to cleavage by convertases due to post-translational modifications of this isoform induced by oxidative stress (Fleitas et al., 2018).

Age-related and pathological alterations in the pro-BDNF/m-BDNF ratio may enhance long-term depression and reduce long-term neuronal plasticity, leading to synaptic dysfunction. Chronic reduction in mature BDNF, which is an important neuroprotective factor in the CNS, leads to neuronal atrophy and death. The age-related shift in the pro-BDNF/m-BDNF ratio plays an important role in the pathogenesis of cognitive deficits. Assessment of this ratio is a promising prognostic marker of cognitive dysfunction, potentially surpassing the accuracy of isolated m-BDNF measurement.

Thus, methodological considerations are of critical importance when interpreting study findings and evaluating the diagnostic potential of BDNF. A comprehensive approach that includes standardization of preanalytical procedures, quality control and assessment of biological sample homogeneity, and the use of highly specific and high-performance assay systems is essential to ensure the validity and reliability of the results obtained.

**FIGURE 1:**
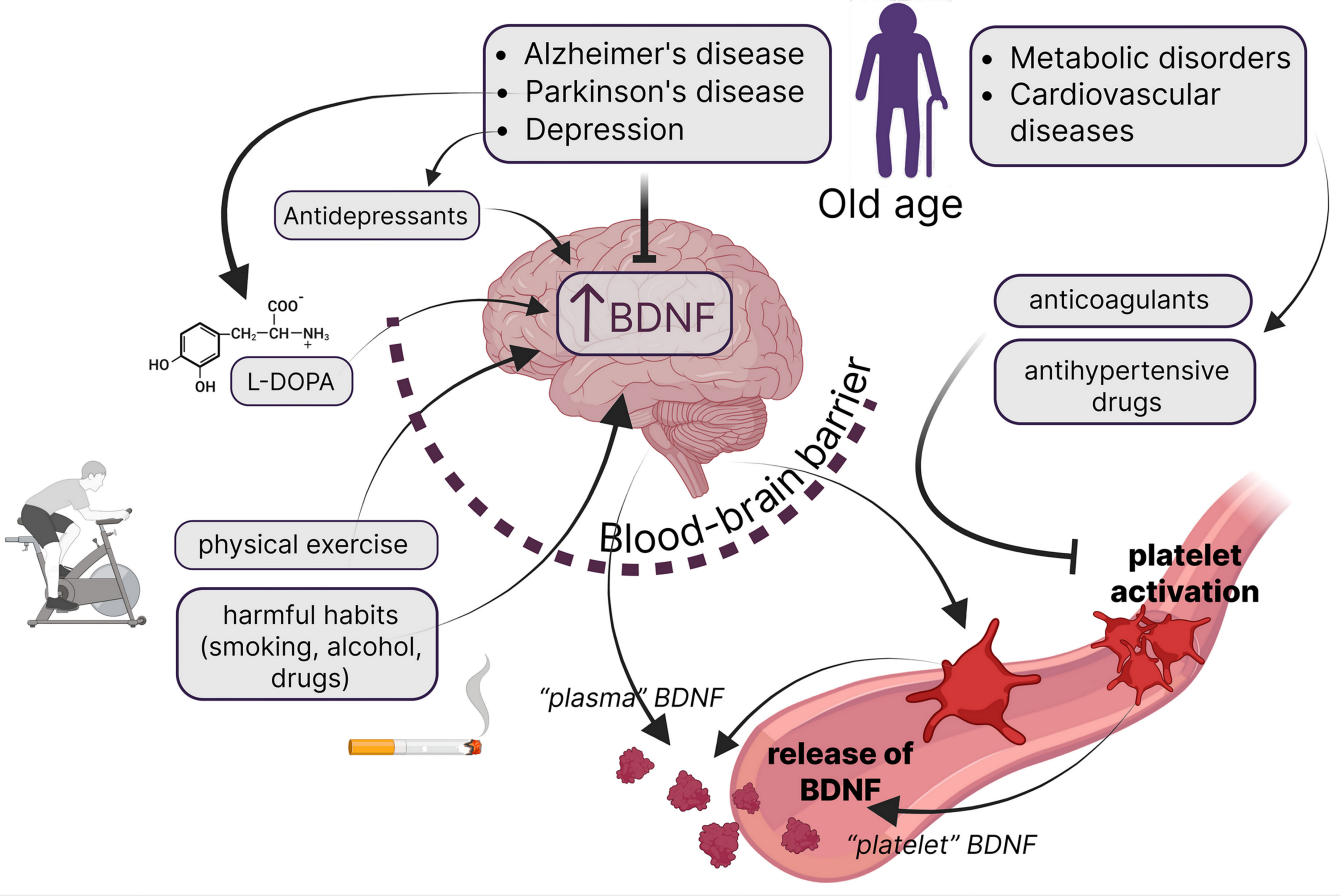
Effect of external factors on circulating BDNF levels (created with BioRender.com).

Failure to adequately control for factors that influence circulating BDNF levels limits both the comparability and generalizability of findings, thereby complicating the assessment of the prognostic value of BDNF (Figure 1). Neurodegenerative disorders and cognitive impairment are multifactorial conditions; therefore, the concentration of a single neurotrophic factor measured in peripheral blood may not fully reflect the complex and dynamic processes occurring within the nervous system. Under these circumstances, alternative approaches may include evaluating the ratio of proBDNF to mature BDNF (Yi et al., 2021) or assessing BDNF in combination with other biomarkers, such as β-amyloid in Alzheimer’s disease (Therriault et al., 2024) or homocysteine (Janel et al., 2017).

## 4 *BDNF* polymorphisms as prognostic markers of age-related cognitive impairment

Given the central role of BDNF in neurogenesis and the maintenance of synaptic plasticity within the hippocampus and cerebral cortex, the potential contribution of *BDNF* gene polymorphisms to age-related cognitive dysfunction has attracted considerable interest. Several studies have demonstrated associations between specific *BDNF* polymorphisms and the development or progression of neurodegenerative disorders.

For example, the polymorphisms rs10501087, rs1491850, and rs11030094 have been associated with a slower progression of Parkinson’s disease, as reflected by a delayed need for symptomatic treatment of motor manifestations (Fischer et al., 2022). In another study, rs11030094 was influenced cognitive test results in auditory and verbal learning (Warburton et al., 2016). Furthermore, rs10501087 and rs1491850 were associated with accelerated hippocampal atrophy in an elderly population, potentially indicating impaired memory retention (Honea et al., 2013). While rs11030094, together with rs1157659 and rs11030108, appeared to contribute to the pronounced cognitive decline observed in Alzheimer’s disease.

One of the most extensively investigated *BDNF* polymorphisms is rs2030324 (C270T). However, evidence regarding its role in neurodegeneration remains inconsistent, a discrepancy frequently attributed to the small sample sizes and heterogeneity of study populations with respect to ethnicity and disease stage. The rs2030324 (*C270T*) variant has been associated with amnestic mild cognitive impairment (aMCI), a transitional state between normal aging and dementia (Xie et al., 2017). Nevertheless, a 2017 meta-analysis encompassing 39 studies across different ethnic populations found no association between the *C270T* polymorphism and Alzheimer’s disease (Shovit and Praveen, 2017). In a Japanese cohort of patients with Alzheimer’s disease, *C270T* was associated with performance on tests of executive function but not memory (Nagata et al., 2011). These findings suggest that *BDNF* polymorphisms may affect specific cognitive domains rather than global cognitive status.

The limited and inconsistent evidence regarding the role of the aforementioned polymorphisms in neurodegeneration and age-related cognitive decline may be related to their localization within non-coding regions of the gene, including intronic variants (rs10501087, rs1491850, rs11030094, and others) and promoter variants such as C270T. This observation suggests that these single-nucleotide polymorphisms (SNPs) may exert their effects primarily through transcriptional or epigenetic mechanisms rather than through direct alterations in protein structure or function.

In contrast, rs6265, located at codon 196 and resulting in a substitution of valine (Val66) with methionine (Met), has attracted considerable attention because of its direct impact on BDNF protein function.

## 5 Val66Met

**FIGURE 2:**
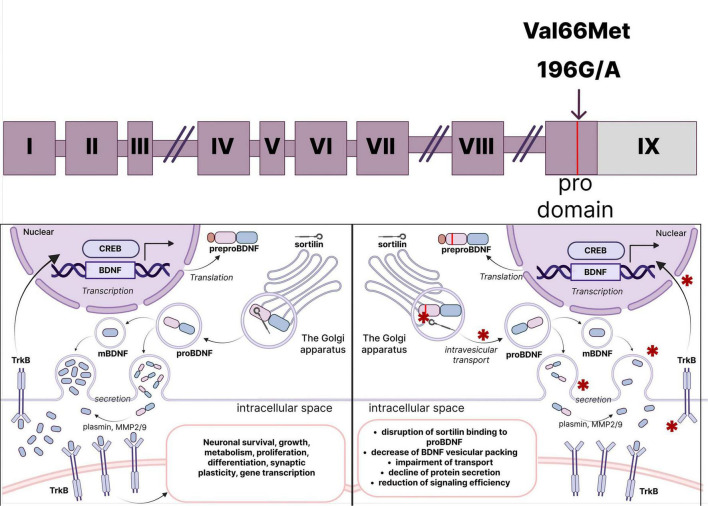
Effects of the BDNF Val66Met polymorphism on BDNF synthesis, secretion, and function (created with BioRender.com).

One of the most extensively studied and common *BDNF* polymorphisms is *Val66Met* (rs6265). This variant is a G>A nucleotide change at position 196 that causes a Val-to-Met substitution at codon 66 in the BDNF prodomain (Figure 2). The substitution affects intracellular trafficking of proBDNF, reduces the secretion of mature BDNF, and impairs dendritic mRNA transport through disruption of the interaction between BDNF and translin (Chiaruttini et al., 2009). In addition, the *Val66Met* substitution alters the sortilin-binding site, thereby reducing vesicular packaging of BDNF, activity-dependent BDNF secretion, and the efficiency of BDNF–TrkB signaling (Baj et al., 2013). At the physiological level, the *Val66Met* variant is associated with reduced metabolic activity in the hippocampus and with decreased volumes of the hippocampus, amygdala, thalamus, and other brain regions.

Particular attention has been directed toward the BDNF *Val66Met* polymorphism because of its potential role in age-related cognitive decline, susceptibility to neurodegenerative diseases, and the rate and severity of disease progression (Ghisletta et al., 2014; Kennedy et al., 2015). For example, several studies have demonstrated that the *Val66Met* polymorphism is associated with an increased risk of progression from subjective cognitive decline to mild cognitive impairment and dementia, as well as with a shorter time to conversion (Boots et al., 2017).

van den Bosch et al. (2021) reported that carriers of the *Val66Met* variant exhibited a more pronounced decline in cognitive performance, including memory, language, executive function, and attention, compared with *Val/Val* homozygotes. Moreover, the presence of the *Val66Met* polymorphism in combination with a positive β-amyloid status was associated with an 8.8-fold increase in the risk of dementia. Notably, because the burden of β-amyloid deposition was similar in carriers of both the G (Val) and A (Met) alleles, the *Val66Met* variant appears to exacerbate rather than directly cause hippocampal atrophy and β-amyloid-related pathology. The combined effect of β-amyloid accumulation and the *Val66Met* polymorphism was associated with impairments in episodic memory and attention, as well as deficits in language and executive function (Lim et al., 2015, 2021).

Franzmeier et al. (2021) demonstrated that carriage of the A (Met) allele increases the susceptibility of the hippocampal–frontal network to the effects of β-amyloid, thereby contributing to cognitive dysfunction during the progression of dementia, particularly Alzheimer’s disease. Similarly, Shen et al. reported that the *Val66Met* variant in older adults was associated with reduced volumes of the entorhinal cortex and posterior cingulate gyrus compared with *Val/Val* homozygotes, although no association with cognitive test performance was observed (Shen et al., 2024).

Furthermore, several studies have suggested that the cognitive decline observed in Met66 carriers may be associated with elevated concentrations of tau protein and its precursor in the cerebrospinal fluid compared with *Val/Val* homozygotes (Thomson et al., 2024; Ciampa et al., 2024). Interestingly, *Val/Val* homozygotes appear to derive greater neuroprotective benefit from physical activity with respect to reductions in p-tau181 levels (Cadwallader et al., 2025). Given that physical exercise increases *BDNF* expression, the *Val66Met* polymorphism may contribute to interindividual variability in the effects of physical activity on age-related cognitive decline.

Nevertheless, the precise impact of the BDNF *Val66Met* polymorphism on cognitive function remains incompletely understood. Some studies have failed to identify an association between the *Val66Met* polymorphism and cognitive impairment (Mandelman and Grigorenko, 2012; Kim et al., 2015), whereas others have suggested that carriage of the A (Met) allele may exert a protective effect on cognitive function during aging (Barha et al., 2019). Schaeverbeke et al. (2021) reported that baseline episodic memory performance, rather than BDNF genotype, influenced the trajectory of age-related episodic memory decline in cognitively healthy older adults. Other studies have suggested that the *Val66Met* polymorphism negatively affects specific cognitive domains, such as memory, without influencing overall cognitive performance (Kambeitz et al., 2012; Toh et al., 2018). Similarly, Harrisberger et al. (2014), as well as their subsequent meta-analysis, found no association between hippocampal volume and the BDNF *Val66Met* polymorphism in healthy individuals.

These inconsistent findings may reflect the influence of factors such as sex, ethnicity, and the presence and clinical phenotype of underlying neurological disorders on the effects of the *Val66Met* polymorphism. For example, approximately 70% of individuals of Asian ancestry carry the BDNF G>A (Met) variant, whereas the frequency of the A (Met) allele in European populations ranges from 30% to 50%. In addition, several investigators have reported unequal distributions of allele carriers within their study cohorts. Such variability in carrier representation may affect sample representativeness, reduce statistical power, and limit the generalizability of findings across different ethnic populations.

Furthermore, the effects of the *Val66Met* polymorphism appear to exhibit substantial sex-related heterogeneity. Some studies have reported an increased risk of dementia exclusively among female carriers of the A (Met) allele (Lim et al., 2021; Fukumoto et al., 2010; Li et al., 2017; Bessi et al., 2020), whereas others have described Met allele carriage as a favorable factor associated with slower cognitive aging in women (Barha et al., 2019). These findings support consideration of the BDNF *Val66Met* polymorphism as a sex-specific risk factor. In a longitudinal study conducted by Lim Y. et al., *Val/Val* homozygotes exhibited a more pronounced decline in memory function following the onset of clinical Alzheimer’s disease, despite evidence indicating that, prior to disease onset, carriers of the G (Val) allele were at lower risk of cognitive impairment than carriers of the A (Met) allele (Lim et al., 2021).

Furthermore, numerous studies have identified *APOE*ε*4* as a risk factor for Alzheimer’s disease and have therefore considered *APOE*ε*4* status when investigating the effects of the BDNF *Val66Met* polymorphism (Ward et al., 2014; Gomar et al., 2016 *;*≪*cps*:*it* > *ref* < /*cps*:*it*≫Cechova et al., 2020). In this context, *Val66Met* has been reported to exert a synergistic effect in combination with *APOE*ε*4* (Lim et al., 2015; Gomar et al., 2016; Ji et al., 2024). Conversely, other studies have found that *Val66Met* has no influence on susceptibility to accelerated cognitive aging or the development of age-related neurodegenerative diseases, irrespective of *APOE*ε*4* status (Zhao et al., 2018).

In addition, *Val66Met* is in linkage disequilibrium with other *BDNF* polymorphisms, including C270T, which may influence gene expression as well as the distribution and function of the protein within neurons. The interaction among these polymorphisms highlights the importance of a comprehensive approach when evaluating BDNF as a prognostic factor.

Despite evidence supporting reduced BDNF secretion associated with Val66Met, this substitution does not appear to affect circulating BDNF levels. Caldieraro et al. (2018) reported that Met carriers exhibited elevated serum BDNF levels. However, most studies have failed to detect an effect of Val66Met on serum (Terracciano et al., 2013; Ramawat et al., 2024; Zhang et al., 2012; Froud et al., 2019) or plasma BDNF concentrations (Chen et al., 2014). These findings, demonstrating the lack of an effect of the Val66Met substitution on circulating BDNF levels, underscore the complexity of extrapolating genetic markers to peripheral protein levels. It is likely that genetically determined features of BDNF secretion into the systemic circulation are outweighed by the dominant influence of uncontrolled factors and individual lifestyle. Analysis of this relationship is considerably hampered by the high variability of confounding variables, necessitating careful revision and refinement.

Thus, the BDNF *Val66Met* polymorphism does not appear to exert a direct effect on the development of age-related cognitive dysfunction and cannot be regarded as an independent risk factor for dementia. However, it may contribute to the progression of existing pathological processes. Because this substitution is associated with impaired intracellular transport and secretion of BDNF, the resulting reduction in the protein’s protective and supportive functions may lead to decreased neuronal plasticity and reduced neuronal resistance to toxic proteins, thereby accelerating neurodegenerative processes. As the brain ages and its chemical and structural reserves decline, the BDNF *Val66Met* polymorphism may contribute to an individual’s genetic susceptibility to neurodegeneration and age-related cognitive impairment. A summary of the effects of various BDNF polymorphisms on cognitive changes, anatomical features, and the development of neurodegenerative processes is presented in Supplementary Table 1 in the Supplementary Materials.

Accurate assessment of the contribution of *BDNF* polymorphisms to neurodegenerative processes and cognitive decline requires carefully selected large study cohorts that account for sex, age, comorbid neurological disorders, and their rigorous classification, as well as standardized approaches to cognitive assessment. Given the complexity of cognitive processes and the incomplete understanding of their underlying mechanisms, determining the significance of a single genetic variant within a complex neural network remains a considerable challenge. Therefore, future studies should take into account:

- the interaction between *BDNF* genetic variants and genes involved in cognitive function;
- the phenotypic and demographic characteristics of study participants.

## 6 Concluding remarks

BDNF is the most important factor involved in maintaining cognitive function in the aging brain and protecting neurons against degeneration associated with age-related diseases. The ability of BDNF to cross the blood–brain barrier, together with the presence of peripheral cellular reservoirs such as platelets, enables the use of peripheral blood as a source for monitoring processes occurring within the brain, which represents a significant advantage for clinical application. In addition, genetic variability within the *BDNF* gene may contribute to individual differences in the rate and severity of cognitive decline, as well as variability in therapeutic response.

However, definitive and reliable evidence regarding the prognostic value of peripheral BDNF has not yet been established. This is attributable to numerous factors, including heterogeneity among study populations with respect to age, sex, health status, and lifestyle, as well as differences in the assessment of comorbidities and cognitive performance. To accurately determine the utility of peripheral BDNF as a biomarker of the onset and progression of age-related cognitive impairment and neurodegenerative processes, standardized studies that account for these factors are required.
