# Supplementary material for: The prognostic potential of circulating BDNF levels and its polymorphisms in age-related cognitive impairment and neurodegeneration
Source: Front Aging Neurosci. 2026 Jul 27;18:1901527. doi: 10.3389/fnagi.2026.1901527 (PMC13454533; doi:10.3389/fnagi.2026.1901527)
Supplement: Supplementary file 1 [file Table_1.pdf]

Supplementary Material

Supplementary Table 1. Associations of *BDNF* polymorphisms with cognitive changes, brain morphometry, and neurodegenerative outcomes

**Impact on cognitive test performance**								
**SNP of *BDNF***	**Population**	**Number of participants**	**Composition of cases**			**Allelic Variant**	**Effect**	**Reference**
rs6265			Val/Val	Met carries				
				Val/Met	Met/Met			
	Cognitively intact	1023	693 (67.7%)	296 (28.9%)	34 (3.3%)	GA (Val/Met)	Sharp decline in verbal learning and memory scores	Boots, E. et al (2017)
	Cognitively intact	398	255 (64%)	126 (31.6%)	17 (4.3%)		Sharp decline in global cognitive functions, memory, speech, executive functions, and attention, coupled with the highest risk of dementia	van den Bosch et al. (2021)
	MCI and AD	384	246 (64%)	138 (36%)			Reduced episodic memory scores and a 1.5-fold higher risk of mild cognitive impairment and Alzheimer's disease	Lim, Yet al. (2021)
	patients with Parkinson's disease	175	117 (66.8%)	58 (33.2%)			Association with more pronounced cognitive impairment	Altmann, V.et al. (2016)
	Healthy adults	314	191 (60.8%)	111 (35.4)	12 (3.8%)		No effect on episodic memory tests without a positive beta-amyloid test	Lim, Yet al. (2015)
	Healthy adults	444	322 (72.6)	121 (27.4)			No effect on working memory, episodic memory, or executive function	Thow, M. E. et al. (2017)
	a meta-analysis of healthy people and people with	7095	data not described				Had no effect on global cognitive abilities, memory scores,	Mandelman, S.D. and

	various mental illnesses						executive functions, or measures of cognitive fluency	Grigorenko, E.L. (2012)
	Cognitively Normal (ADNI +ADRC)	422	287 (68%)	126 (30%)	9 (2%)		Was not associated with memory performance or associative learning	Kim et al., 2015
	Early-Stage Dementia (ADNI + ADRC)	558	363 (65%)	178 (32%)	17 (3%)			
	Healthy adults	180	93 (51.7%)	87 (48.3%)			No significant effect on episodic memory	Schaeverbeke et al., 2021
	Elderly participants without dementia	74	43 (58.1%)	28 (37.8%)	3 (4.1%)	AA (Val/Val)	greater decline in executive function scores	Bessi et al., 2020
rs11030094	Patients with epilepsy	72	GG	GA	AA	GA	Affected performance on the auditory verbal learning test	Warburton et al., 2016
			28 (38.8%)	28 (38.8%)	11 (15.3%)			
rs2030324			TT	TC	CC			
		78	25 (32%)	37 (47.4%)	16 (20.5%)	TC		
rs1491850		82	24 (29.3%)	44 (53.6%)	14 (17%)	TC		
**Effects on brain anatomical structures**								
rs6265			Val/Val	Met carries				
				Val/Met	Met/Met			
	elderly people	108	64 (59.3%)	39 (36.1%)	5 (4.6%)	GA (Val/Met)	Reduced volume of the entorhinal cortex and the posterior cingulate gyrus	Shen et al., 2024
	healthyyoung	643	413 (64.2%)	204 (31.7%)	26 (4%)		Had no effect on hippocampal volume	Harrisberger et al., 2014
	ADNI project	536	67%	30%	2%		Had no effect on hippocampal volume	Kim et al., 2015
	Knight ADRC	302	69%	30%	2%			
rs11030094	Moderate cognitive impairment, MCI, AD	329	GG	GA	AA	GA	Increased hippocampal atroph	Honea et al., 2013
			97 (29.5%)	160 (48.6%)	72 (21.9%)			
rs10501087			TT	TC	CC			

			217 (66%)	112 (34%)		TC		
rs1491850			109 (33.1%)	155 (42.1%)	65 (19.6%)	TC		
**Impact on pathological factors**								
rs6265			Val/Val	Met carries				
				Val/Met	Met/Met			
	ADAD-causing mutations; older adults	378	data not described			GA (Val/Met)	Pronounced reduction in hippocampal–medial–frontal connectivity and deterioration of cognitive function	Franzmeier et al., 2021
	patients with Alzheimer's disease with normal cognitive function or MCI, as assessed by the scale MMSE	286	192 (67.1 %)	94 (32.9%)			Elevated levels of tau protein and its precursor in the cerebrospinal fluid, accompanied by cognitive decline	Thomson et al., 2024; Ciampa et al., 2023
	elderly people with normal cognitive function, assessed by the MMSE scale, Wechsler Memory Scale and GDR	321	208 (65%)	100 (31%)	13 (4%)		Pronounced tau accumulation in the temporal lobe	Ciampa et al., 2023
	Older adults without dementia for Cognitive Health study (BrANCH)	180	105 (58.3%)	64 (35.6%)	11 (6.1%)	Val/Val	Marked positive effect of physical activity on tau reduction	Cadwallader et al., 2015

CDR - Clinical Dementia Rating
AD - Alzheimer's disease
MCI - Mild Cognitive Impairment
ADNI - Alzheimer’s Disease Neuroimaing Initiative
ADRC - Alzheimer Disease Research Center
MMSE - Mini-Mental State Examination
